# Significant incidents during X-ray exposures in humans – assessment and findings of the Federal Office for Radiation Protection

**DOI:** 10.1055/a-2339-3684

**Published:** 2024-09-11

**Authors:** Katharina Stella Winter, Monika Schweden, Gunnar Brix, Erik Mille

**Affiliations:** 1 84634 Generic aspects of medical radiation protection, Federal Office for Radiation Protection Neuherberg, Oberschleißheim, Germany

**Keywords:** significant incidents, incident reporting system, radiation safety, diagnostic radiology, interventional procedures

## Abstract

**Background:**

In Germany, significant incidents must be reported to the competent authority since 12/31/2018 (section 108 Radiation Protection Ordinance (StrlSchV)). After assessment and evaluation of the reports, the competent authority submits the relevant information via a web-based reporting system (BeVoMed) to the Federal Office for Radiation Protection (BfS), which publishes the results derived therefrom. The present paper evaluates significant incidents related to X-ray exposures on humans.

**Methods:**

All reports on incidents in X-ray diagnostics and interventional radiology between 01/2019 and 10/2023, which were completed with detailed information until the reporting day (31/10/2023), were included. The following aspects were statistically evaluated: classification as an incident (section 1 subsection (22) StrlSchV), significance (section 108 StrSchV), classification to Annex 14 StrlSchV, assignment to the forms of care in the German healthcare system, and development of the reporting frequency over time. Furthermore, the content of the reports was systematically evaluated with regard to conspicuous clusters and typical problems.

**Results and Conclusion:**

Until the reporting day, 383 reports (355 completed) were received. 252 reports (228 in X-ray diagnostics, 24 in interventional radiology) referred to significant incidents and were included in the detailed evaluation. Reporting frequency increased in X-ray diagnostics, whereas there was no trend in interventional radiology. Most of the significant incidents concerned examinations on an individual person (category I, criterion 2a or category II, criteria 2a and 3a) in the in-patient sector – especially in maximum care hospitals. Frequent errors concerned the inappropriate choice of parameters/protocols or were related to the administration of a contrast agent. Despite the overall positive trend, the establishment of awareness and error culture remains challenging.

**Key Points:**

- In X-ray diagnostics, the reporting frequency of significant incidents increases.
- Most incidents concerned CT examinations in the in-patient sector.
- Main problems were selection of protocols/settings, contrast administration, and bolus-tracking.
- Uncertainties remain about the classification of an incident as significant and notifiable.

**Citation Format:**

- Winter KS, Schweden M, Brix G et al. Significant incidents during X-ray exposures in humans – assessment and findings of the Federal Office for Radiation Protection. Rofo 2025; 197: 154–162

## Introduction

The use of ionising radiation in humans is an essential part of radiological diagnostic examinations and therapy. The absolute number of examinations, especially the number of computed tomography (CT) procedures, has increased dramatically in recent decades 1 . But this increase has also intensified the risk of (near) mistakes or accidents which could result in injury to patients or staff. To avoid mistakes and improve the radiation exposure safety, in the context of a revision of the German radiation protection law, lawmakers in Germany introduced a reporting requirement for significant events/incidents occurring during diagnostic radiological procedures in humans in accordance with section 90 of the Radiation Protection Act (Strahlenschutzgesetz, StrlSchG) and section 108 of the Radiation Protection Ordinance (Strahlenschutzverordnung, StrlSchV) on 31 December 2018.

According to the new rules, the radiation protection executive (Strahlenschutzverantwortlicher, SSV) of an institution must immediately report the occurrence of a significant incident to the competent authority; a complete summary report must be presented within six months of the event. An incident as defined in section 1 subsection 22 of the StrlSchV is an event which occurred during a planned exposure setting which has resulted or could have resulted or could result in unplanned exposure. During diagnostic radiological procedures in humans, section 108 subsection 1 states that an incident is particularly significant if it meets one of the criteria of Annex 14 of the StrlSchV.

For legal reasons specific to the German system and its federal executive administration, significant events must be reported to the regional radiation protection competent supervisory authorities, after which these regional authorities can and, where necessary, must take action in accordance with section 110 of the StrlSchV. The competent state authorities evaluate the incoming reported events and pass on relevant information about these events in pseudonymised form to the German Federal Office for Radiation Protection (Bundesamt für Strahlenschutz, BfS). The BfS is the central body which collects and evaluates reported significant events in terms of their relevance for other operators across Germany in accordance with radiation protection and patient safety. A detailed description of the structure of the reporting system and the offices/authorities involved is given in an article by Brix et al. 2 . The BfS publishes the results of its evaluations in annual reports 3 . If it is technically expedient, brief information about specific occurrences are provided to affected specialist circles on a password-protected area of the BfS homepage 4 .

The BfS statistically evaluates the number and type of reported events as well as evaluating the contents of reported incidents with regards to typical mistakes and possible sources of error. The aim is to avoid the same or similar events occurring in other institutions. This study summarises the statistical evaluations and lessons learned from significant events which occurred in the context of X-ray examinations in humans for the period from January 2019 to October 2023.

## Method

The reports sent to the central body are processed using a web-based IT system established by the BfS to deal with significant incidents in medicine (bedeutsame Vorkommnisse in der Medizin, “BeVoMed”). This system gathers the information forwarded by the competent authorities about significant events and then processes and evaluates the information. The BeVoMed system has been revised several times since it was first introduced in 2019 and now provides better analysis options not just for the BfS but also for competent authorities. For this study, all reported events involving X-ray procedures were reviewed systematically and in context. Based on the experience accumulated since 2019, in a few cases this led to a specialist re-assessment, meaning that the figures presented hereinafter may deviate slightly from the figures given in the annual reports for 2019 – 2022.

All reports on diagnostic and interventional X-ray procedures passed on to the BfS between January 2019 and October 2023 for which a closing report was available by the cut-off date of 31 October 2023 were evaluated. A statistical evaluation was first carried out. To do this, all received reports were classified with regards to

- their categorisation as an event/incident as such (section 1 subsection 22 StrlSchV),
- their significance (section 108 or Annex 14 of the StrlSchV),
- the respective reporting criteria given in Annex 14 StrlSchV ( Table 1 ) or the significance outside of Annex 14, and
- the classification of the event with regards to the location where it occurred in the German healthcare system (i.e., GP practice, joint practice, primary care hospital, secondary care hospital, tertiary care hospital, community health centre, other).

**Table:** **Table 1** Criteria for the significance of incidents according to Annex 14 StrlSchV that are relevant for X-ray exposures.

**Category I**	**Examinations with ionising radiation – without interventions**	
**Number 1**	**Relating to a group of persons**	
**Number 2**	**Relating to an individual person**	
	**A**	CTDI _vol_ of computed tomography of the brain > 120 mGy or of the body > 80 mGy, DAP of X-ray imaging > 20,000 cGy cm ^2^
	**B**	Repetition of a procedure resulting in the dose being exceeded
	**C**	Misidentified person resulting in the dose being exceeded
	**D**	Unexpected deterministic effect
**Category II**	**Interventions**	
**Number 1**	**Relating to a group of persons**	
**Number 2**	**Relating to an individual person if the intervention takes place for the purpose of examining the person**	
	**A**	DAP > 20,000 cGy cm ^2^
	**B**	Repetition of a procedure resulting in the dose being exceeded
	**C**	Misidentified person
	**D**	Unexpected deterministic effect
**Number 3**	**Relating to an individual person if the intervention is carried out for the purpose of treating the person**	
	**A**	Deterministic skin damage with DAP > 50,000 cGy cm ^2^
	**B**	Misidentified person or body part
	**C**	Unexpected deterministic effect
**Category V**	**Carers and comforters in accordance with section 2 subsection (8) no. 3 of the StrlSchG**	
**Category VI**	**Use of ionising radiation or radioactive substances on humans for the purpose of medical research**	
**Category VII**	**Incidents in which exposure almost occurred**	

For events classified as significant, the aspects listed above were grouped together for every quarter of the evaluation period and analysed statistically to assess developments over time since the introduction of the reporting obligation.

A final systematic evaluation of the contents of reports identified the most commonly occurring problems and the underlying causes in detail.

## Results

### Statistical evaluation

By the cut-off date (31 October 2023) a total of 383 initial reports on diagnostic and interventional X-ray procedures had been forwarded to the BfS, of which 355 reports (278 on diagnostic X-ray procedures and 77 on interventional procedures) had been brought to a final conclusion. The BfS was not able to understand why 82 reports were flagged up as an event, and a further 21 reports were not considered significant by the BfS. This left a total of 252 significant events (228 diagnostic and 24 interventional X-ray procedures) which were included in our further analysis ( Table 2 ).

**Table:** **Table 2** Distribution of all completed reports (as of 31/10/2023) on diagnostic and interventional X-ray exposures stratified by the criteria given in Annex 14 Radiation Protection Ordinance (StrlSchV), according to their classification as an incident (section 1 subsection (22) StrlSchV) or as significant (section 108 StrlSchV).

Category, criterion		Total	Significant	Not significant	Not an event
**I. Diagnostic**		**267**	**223**	**15**	**29**
No. 1		17	17	–	–
No. 2	a)	219	190	–	29
	b)	16	15	1	–
	c)	15	1	14	–
	d)	**–**	–	–	–
Outside Annex 14		**10**	**5**	**5**	–
**II. Interventional**		**65**	**18**	**–**	**47**
No. 1		1	1	–	–
No. 2	a)	55	10	–	45
	b)	–	–	–	–
	c)	–	–	–	–
	d)	–	–	–	–
No. 3	a)	7	5	–	2
	b)	–	–	–	–
	c)	2	2	–	–
Outside Annex 14	CT interventions	**11**	**5**	–	**6**
	Other	**1**	**1**	–	**–**
**VI. Medical research**		**1**	**–**	**1**	**–**
**Total**		**355**	**252**	**21**	**82**

**Fig. 1:**
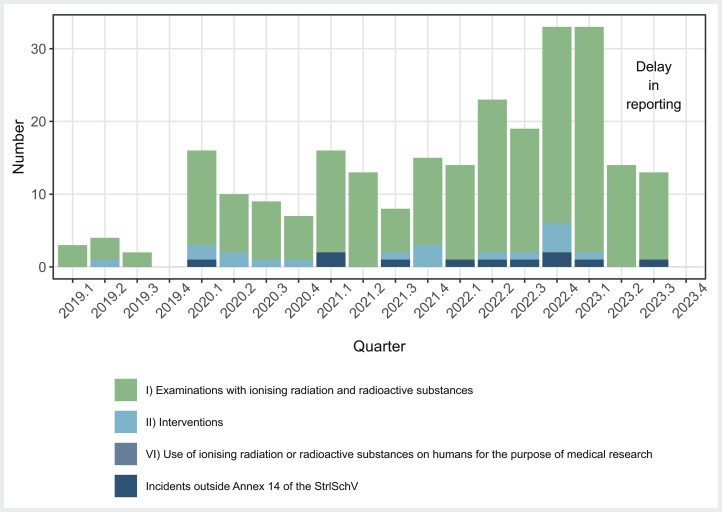
Quarterly presentation of the frequency of significant incidents concerning diagnostic and interventional X-ray exposures stratified by the criteria given in Annex 14 StrlSchV.

With regards to diagnostic X-ray procedures, the number of reported significant events increased significantly until 2022; this trend continued in 2023, although it should be noted that reliable figures for the year 2023 are only available up until the end of the first quarter, due to the delay in reporting in accordance with the deadlines for reporting events under section 108 StrlSchV ( Table 3 ). A correlation analysis using the quarterly data showed a weakly positive but significant increase. No intra-year trend for the quarters was found ( Fig. 1 ).

**Table:** **Table 3** Distribution of the significant incidents (as of 31/10/2023) concerning diagnostic and interventional X-ray exposures stratified by the criteria given in Annex 14 StrlSchV over the years 2019–2023.

Category, criterion		2019	2020	2021	2022	2023*
**I. Diagnostic**		**8**	**35**	**45**	**78**	**57**
No. 1		3	7	1	4	2
No. 2	a)	5	24	38	69	54
	b)	–	3	6	5	1
	c)	–	1	–	–	–
	d)	–	–	–	–	–
Outside Annex 14		**–**	**1**	**2**	**1**	**1**
**II. Interventional**		**1**	**6**	**4**	**6**	**1**
No. 1		–	1	–	–	–
No. 2	a)	1	2	4	2	1
	b)	–	–	–	–	–
	c)	–	–	–	–	–
	d)	–	–	–	–	–
No. 3	a)	–	1	–	4	–
	b)	–	–	–	–	–
	c)	–	2	–	–	–
Outside Annex 14	CT interventions	**–**	**–**	**1**	**3**	**1**
	Other	**–**	**–**	**–**	**1**	**–**
**Total**		**9**	**42**	**52**	**89**	**60**
*Up until 31 October 2023.						

Most reports related to reporting thresholds for individual persons being exceeded during diagnostic CT (category I, criterion 2a), followed by thresholds being exceeded in groups of patients due to similar CT examinations (category I, criterion 1) and repeat procedures (category I, criterion 2b) being carried out. There was only one report of a misidentified person which resulted in the maximum dose being exceeded (category I, criterion 2c). No deterministic effect was reported and none would have been expected for this type of examination (category I, criterion 2d). Only a few reported events occurred in the context of interventional X-ray procedures ( Table 3 and Fig. 1 ). In these cases, most of the events occurred when the reporting threshold for individual patients was exceeded during diagnostic (category II, criterion 2a) or therapeutic interventions (category II, criterion 3a). There were a few individual reports about events classified as significant outside the criteria listed in Annex 14 ( Table 2 and Fig. 1 ). This particularly applied to reports for CT procedures.

**Fig. 2:**
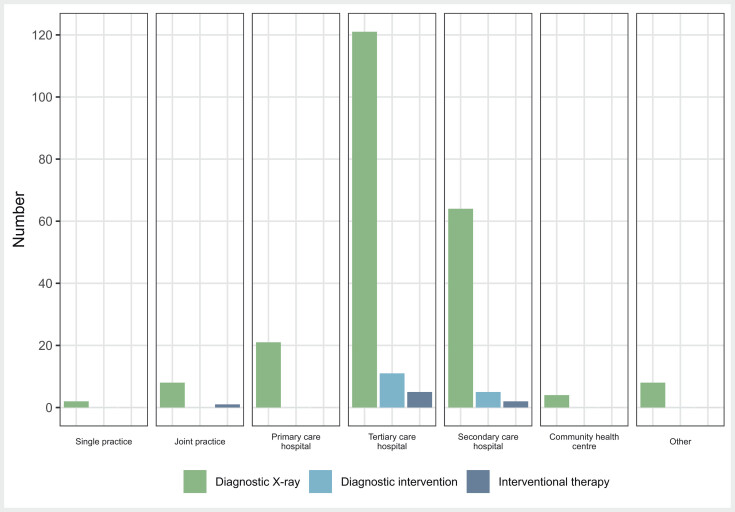
Number of significant incidents assigned to the forms of care in the German healthcare system and the types of applications of X-ray exposures.

With regards to the different types of healthcare institutions, the frequency with which incidents were reported varied greatly. Tertiary care hospitals were most likely to report an event involving a diagnostic X-ray procedure, followed by secondary care and primary care hospitals. They were also the only healthcare institutions which reported incidents involving interventional X-ray procedures, although again most reports came from tertiary care hospitals. The fewest reports came from GP practices, joint practices, or community health centres ( Fig. 2 ).

### Systematic evaluation

Reports about diagnostic X-ray incidents sent to the BfS which the BfS did not consider to be events which merited reporting only involved diagnostic CT procedures carried out in individual patients. They included eight reports of CT perfusion imaging of the head for stroke evaluation. Reported diagnostic X-ray procedures which were not considered significant included 14 cases of misidentified persons, none of which exceeded the reporting threshold (category I, criterion 2c). The overwhelming majority of reported interventional X-ray events were diagnostic angiographies carried out in individual patients, where no inadvertent exposure occurred, although the reporting threshold was exceeded.

A review of significant incidents showed that one problem area was incorrect choice of parameters or protocol which led to reporting thresholds being exceeded. In these cases, patient-specific amendments were made to standard settings which were not justified. In a few cases, automatic adjustment of the tube current and tube voltage resulted in reportable overexposure. In just a few examples, the system’s automatic modulation of the two parameters was disturbed by a sandbag used to position the patient or by a change in the position of the arm after the survey radiograph had been carried out.

There was also a higher incidence of events which involved problems or mistakes which occurred while administering the contrast medium. Especially in CT angiographies which used bolus tracking, reported incidents included no administration or improper administration of the contrast agent, e.g., due to a defective contrast medium pump, an improperly connected tube, a closed three-way valve, or extravasation. Such incidents along with non-optimal selections of parameters for bolus tracking, a delay in or lack of rapid rise in the contrast agent which consequently prolonged the tracking time resulted in reporting thresholds being exceeded. Sometimes an examination had to be repeated once or even several times, which again meant that the sum of the radiation exposures also exceeded the reporting threshold.

Because the number of reports was limited (outside the criteria listed in Annex 14 of the StrlSchV), no specific problem areas were identified for interventional CT procedures.

## Discussion

Since the reporting system for significant events was first introduced, there has been an annual increase in reports with regards to diagnostic X-ray procedures; however, there are only sporadic and relatively few incident reports for interventional X-ray procedures. Given the fact that about 12 million CT scans are carried out annually in Germany 1 , the number of significant and therefore reportable events would be expected to be higher. A research project devised in 2018, which has been finalised in 2020, and curated by the BfS showed that an estimated 1000 significant events for both types of procedures would be expected to occur annually in hospitals alone 5 .

On the other hand, some reported events were not considered by the BfS to constitute an event (section 1 subsection 22 of the StrlSchV) or were as not categorised as significant (section 108, Annex 14 of the StrlSchV). For example, higher doses were administered during CT examinations of obese patients to achieve the required diagnostic imaging quality or the reporting threshold was deliberately exceeded if this made it possible to achieve a diagnostic or therapeutic objective during complex angiography interventions. It can be concluded from this that there is still a lot of uncertainty on the part of radiation protection executives, both about the definition of a reportable event and about its significance. To be categorised as a reportable event, the incident must also involve unintended exposure, i.e., exposure which was not prospectively planned in the context of the individual patient’s indication where the patient was expected to be exposed to radiation. Consequently, a *prospective* planned procedure with an indication justifying radiation exposure (section 83 subsection 3 of the StrlSchG) carried out according to standard procedures does not constitute a reportable event even if the respective reporting threshold is exceeded, e.g., due to the patient’s weight or height. This especially applies to incident reports of diagnostic interventional procedures reporting higher radiation doses where exposure to a higher dose was intended.

It also appears that there are still misunderstandings and ambiguities about what constitutes a significant event. As section 108 of the StrlSchV refers “in particular” to the criteria listed in Annex 14 of the StrlSchV, these criteria are not considered a definitive list. This leaves a certain leeway when evaluating an event for which no explicit reporting criterion is given in Annex 14. Events were also supposed to be reported as significant if a repetition of the incident by another user could be avoided or if a repetition of the incident during similar procedures could be avoided by providing appropriate directions. The BfS has set up a relevant webpage to clarify the isssue and answer frequently asked questions (FAQ) about the interpretation of Annex 14 of the StrlSchV 6 .

Significant events during X-ray examinations occurred mostly in the context of diagnostic CT scans of individual patients (category I, criterion 2a), followed by CT scans in groups of persons (category I, criterion 1). For example, in one case, CT examinations of the bony lumbar spine were carried out in 25 patients using a protocol which was designed for examinations of intervertebral discs 7 . CT examinations are quite common and are also explicitly referred to in Annex 14 of the StrlSchV due to the relatively high exposure occurring during examination, whereas very common procedures such as projection radiography and digital volume tomography of the teeth and the jaw have been excluded from the list due to the very low exposure during these procedures (see the justification in the StrlSchV 8 and for further explanations, Brix et al. 2 ).

Most incident reports about interventional procedures involved diagnostic angiographies, followed by vascular procedures. As Annex 14 did not define any criteria with regards to the significance of events occurring during CT procedures, in each case the decision whether an event should be categorised as significant is up to the radiation protection executive, ideally after consultation with the competent authority.

Most incident reports relating to diagnostic X-ray procedures came from tertiary care hospitals. This can be explained by the fact that these institutions often carry out complex diagnostic and therapeutic radiation procedures, and the institutions usually also have established and detailed quality assurance systems. In contrast, there were very few incident reports from non-hospital-based practices for both diagnostic and interventional X-ray procedures. With regard to interventional procedures, this is due to the fact that, overall, such procedures are not often carried out as outpatient procedures. Nevertheless, significantly more incident reports could be expected from this sector. Beyond that, this could indicate that the new Radiation Protection Act ist not generelly well known yet and consequently, there is only limited awareness that certain events need to be reported.

The analysis of the contents in the reports sent to the BfS showed that the choice of the right protocols and setting the optimal protocol parameters for examinations was a problematic area. Inexperienced adjustments to the tube current and voltage can lead to higher dose exposures which exceed the reporting threshold. If the presettings are unsatisfactory, especially if the standard protocols are inadequate, a succession of patients could be affected by higher exposures. To avoid this, the standard protocols of every institution must be reviewed regularly in cooperation with a medical physics expert (cf. section 131 StrlSchV). In principle, dose management systems are a useful tool to spot unjustified radiation exposure.

The observed increase in the number of events occurring in connection with the administration of contrast agents during CT examinations was largely the result of bolus tracking. Delayed appearance of the contrast medium or no rapid rise of the contrast medium extends tracking times, resulting in unintended CTDI _vol_ values of more than 80 mGy which need to be reported. The radiation hygiene relevance of such exposures in a single or a few CT layers can, of course, not be compared with when the CTDI _vol_ value is exceeded across a larger area of the body such as the abdomen. Nevertheless, even in these cases, there is an obligation to optimise radiation protection, both with regards to the tube current used and, in particiular, with regards to the image repeat rate and the duration of tracking. In the context of radiation protection, it is also worth critically reviewing whether every procedure requires bolus tracking. This should be considered when making a decision about an indication which would justify the use of bolus tracking.

It is proposed that clinical institutions should establish a positive failure culture which does not shame and blame. The BfS is of the opinion that the basic prerequisite for optimising processes is a functioning local quality management system which meets the requirements set out in section 105 of the StrlSchV. Useful measures include implementing dose management systems or compiling work procedures. Likewise, as mentioned previously, regular optimisation of protocols for the devices used in an institution is recommended. If the specific clinical situation requires a deviation from established work processes and protocols (which may be fully justified in individual cases), then the resulting higher potential risk must be considered. As part of patient safety requirements, it is important to ensure that staffing levels are sufficient and that the appropriate safety precautions are functioning 9 . The changes in the numbers of reported cases since the introduction of the obligation to report significant events points to an increased prevalence of quality assurance systems which identify and prevent events at a local level, an increased awareness of problems, and a general acceptance of the obligation to report events. The obligatory cooperation with medical physics experts (section 131 subsection 2 no. 3 and no. 4 StrlSchV) has played a role, especially since the transition period ended (sections 198 and 200 StrlSchG), the reporting obligation now also covers existing equipment.

Compared to the numbers reported internationally, the frequency with which significant events are reported in Germany is, relative to Germany’s population, lower than that of Ireland 10 , Australia 11 or Switzerland 12 . But as the criteria for the significance of events and the organisation of healthcare systems in these countries differ from the situation in Germany, direct comparisons are only possible to a limited extent. The most commonly reported events in the above listed countries also occurred in the context of diagnostic CT procedures. The reasons behind patients being exposed to higher doses varied: in Ireland, it was largely due to human error, in Australia it was caused by unnecessary or unintended examinations and failures of technical devices, and in Switzerland it was due to misidentified persons, inadequate examination protocols, and unintended repeat examinations. As was the case in Germany, only a few incidents were reported in the context of interventional procedures. The Australian Radiation Protection and Nuclear Safety Agency also includes reported angiographies with higher dose exposures due to factors which could not be changed such as complexity or obesity.

There is still some confusion on the part of both radiation protection executives and the regional competent authorities with regards to the definition of events/incidents and their significance. The specified reporting channels in Germany require the radiation protection executive to report a significant event to the competent authority which then reviews the incident report and, if a significant event is found to have occurred, passes on the technically relevant information to the BfS. But as these competent authorities have a certain latitude in what they report and as not every reported event is really a reportable incident, and not every reported event is significant, the number of events reported by radiation protection executives and the number of events ultimately reported to the BfS differ. As the reporting system was set up as part of the radiation protection oversight system, it cannot be precluded that reports are not being sent for fear of negative consequences. Unfortunately, due to how the system is set up, the BfS has no specific figures on either of these issues.

In summary, in recent years there have been positive developments in the reporting system for significant events occurring in the context of the application of radiation doses to humans during X-ray procedures, which indicate the increasing implementation of a positive failure culture. But there still seems to be a lack of clarity with regard to the classification of an event as a reportable incident and the significance of reported events. The goal of all persons and agencies involved should be to continue to improve radiation safety in Germany. For this, continued improvement and strengthening of the awareness of the problem together with a positive failure culture in all professional and occupational groups is key.
