# Supplementary material for: Significant incidents during X-ray exposures in humans – assessment and findings of the Federal Office for Radiation Protection
Source: Rofo. 2024 Sep 11;197(2):154–62. doi: 10.1055/a-2339-3684 (PMC11761057; doi:10.1055/a-2339-3684)
Supplement: Related texts

# Review article: Bedeutsame Vorkommnisse bei Röntgenanwendungen am Menschen – Auswertungen und Erkenntnisse des BfS

**Keywords:** significant incidents, incident reporting system, radiation safety, diagnostic radiology, interventional procedures

## Abstract

**Hintergrund:**

In Deutschland besteht seit dem 31.12.2018 eine Meldepflicht für bedeutsame Vorkommnisse gegenüber der jeweils zuständigen Behörde (§ 108 Strahlenschutzverordnung (StrlSchV)). Diese übermitteln nach Prüfung und Bewertung der Meldung relevante Informationen über ein webbasiertes Meldesystem (BeVoMed) an das Bundesamt für Strahlenschutz (BfS). Das BfS veröffentlicht daraus gewonnene Erkenntnisse. Die vorliegende Arbeit untersucht bedeutsame Vorkommnisse bei Röntgenanwendungen am Menschen.

**Methode:**

Die vorliegende Auswertung berücksichtigt alle Meldungen zu Röntgenuntersuchungen und -interventionen zwischen 01/2019 und 10/2023, für welche bis zum Stichtag (31.10.2023) eine Abschlussmeldung mit Detailinformationen vorlag. Statistisch ausgewertet wurden: Einordnung als Vorkommnis (§ 1 Abs 22 StrlSchV), Bedeutsamkeit (§ 108 StrlSchV), Klassifikation nach Anlage 14 StrlSchV, Zuordnung zu den Versorgungsformen im deutschen Gesundheitssystem sowie die zeitliche Entwicklung der Meldefrequenz über den Auswertewertezeitraum. Zudem wurden die Meldungsinhalte systematisch bezüglich auffälliger Häufungen und typischer Probleme ausgewertet.

**Ergebnisse und Schlussfolgerung:**

Zum Stichtag lagen dem BfS 383 Meldungen (355 abgeschlossene) vor. 252 Meldungen (228 zur Röntgendiagnostik, 24 zur Röntgenintervention) betrafen bedeutsame Vorkommnisse und gingen in die detaillierte Auswertung ein. Für die Röntgendiagnostik zeigt sich ein Anstieg der Meldefrequenz über den Auswertezeitraum, bezüglich der Intervention war kein Trend erkennbar. Die meisten bedeutsamen Vorkommnisse betrafen Anwendungen bei Einzelpersonen (Kategorie I, Kriterium 2a; Kategorie II, Kriterium 2a/3a) im stationären Bereich – insbesondere in Krankenhäusern der Maximalversorgung. Ein Problemfeld bildete die fehlerhafte Parameter-/Protokollanwahl, daneben kam es zu Problemen und Fehlern in Zusammenhang mit der Kontrastmittelgabe. Trotz der insgesamt positiven Entwicklung stellt die weitere Etablierung eines angemessenen Problembewusstseins und einer entsprechenden Fehlerkultur eine Herausforderung dar.

**Kernaussagen:**

- In der Röntgendiagnostik nimmt die Meldefrequenz bedeutsamer Vorkommnisse zu.
- Die meisten Vorkommnisse betrafen CT-Untersuchungen aus dem stationären Bereich.
- Parameter-/Protokollwahl und Kontrastmittelgabe sind Hauptproblemfelder, letzteres häufig im Zusammenhang mit Bolus-Tracking.
- Bei der Einordnung eines Vorkommnisses als bedeutsam und meldepflichtig bestehen noch Unklarheiten.

**Zitierweise:**

- Winter KS, Schweden M, Brix G et al. Significant incidents during X-ray exposures in humans – assessment and findings of the Federal Office for Radiation Protection. Rofo 2025; 197: 154–162

## Einleitung

Der Einsatz ionisierender Strahlung am Menschen bildet einen elementaren Bestandteil der radiologischen Diagnostik und Therapie. So ist die absolute Anzahl an Untersuchungen, insbesondere der Computertomografie (CT), innerhalb der letzten Jahrzehnte drastisch angestiegen 1 . Dadurch erhöht sich jedoch auch das Risiko von (Beinahe-)Fehlern oder Unfällen, welche zu einer Schädigung von Patient*innen oder Personal führen können. Zur Fehlervermeidung und zur Verbesserung der Sicherheit von Strahlenanwendungen hat der Gesetzgeber im Rahmen der Neuregelung des deutschen Strahlenschutzrechts zum 31.12.2018 eine Meldepflicht für bedeutsame Vorkommnisse bei Strahlenanwendungen am Menschen gemäß § 90 Strahlenschutzgesetz (StrlSchG) und § 108 Strahlenschutzverordnung (StrlSchV) eingeführt.

Danach hat der oder die Strahlenschutzverantwortliche (SSV) einer Einrichtung ein bedeutsames Vorkommnis unverzüglich an die zuständige Landesbehörde zu melden; eine vollständige und zusammenfassende Meldung muss binnen sechs Monaten vorgelegt werden. Ein Vorkommnis nach § 1 Abs. 22 der StrlSchV ist ein Ereignis in einer geplanten Expositionssituation, das zu einer unbeabsichtigten Exposition geführt hat, geführt haben könnte oder führen könnte. Bei Strahlenanwendungen am Menschen ist die Bedeutsamkeit eines Vorkommnisses gemäß § 108 Abs. 1 insbesondere dann gegeben, wenn es ein Kriterium der Anlage 14 StrlSchV erfüllt.

Das Meldesystem für bedeutsame Vorkommnisse ist aus rechtssystematischen Gründen im Rahmen der Bundesauftragsverwaltung bei den regionalen Aufsichtsbehörden im Strahlenschutz verortet, wodurch diese nach § 110 StrlSchV aufsichtlich tätig werden können und ggf. müssen. Die zuständigen Landesbehörden bewerten die Vorkommnismeldungen und leiten relevante Informationen dazu in pseudonymisierter Form an das Bundesamt für Strahlenschutz (BfS) weiter. Im Sinne des Strahlenschutzes und der Patient*innensicherheit sammelt und wertet das BfS als zentrale Stelle die gemeldeten bedeutsamen Vorkommnisse hinsichtlich ihrer Relevanz für andere Betreiber bundesweit aus. Eine ausführliche Beschreibung der Struktur des Meldesystems und der beteiligten Akteure sind in einer Publikation von Brix et al. 2 zu finden. Das BfS veröffentlicht die Ergebnisse seiner Auswertungen u.a. in Form von Jahresberichten 3 . Für die betroffenen Fachkreise werden darüber hinaus, wenn fachlich sinnvoll, kurzfristige Informationen in einem passwortgeschützten Bereich der BfS-Homepage bereitgestellt 4 .

Konkret wertet das BfS sowohl statistisch die Anzahl und Art der gemeldeten Vorkommisse als auch die Meldungsinhalte in Bezug auf typische Fehler sowie mögliche Fehlerquellen mit dem Ziel aus, gleiche oder ähnliche Vorkommnisse in anderen Einrichtungen zu vermeiden. Die vorliegende Arbeit fasst die statistischen Auswertungen und gewonnenen Erkenntnisse zu bedeutsamen Vorkommnissen bei Röntgenanwendungen am Menschen für den Zeitraum 01/2019 bis 10/2023 zusammen.

## Methode

Die Bearbeitung der eingehenden Meldungen in der zentralen Stelle erfolgte mittels eines vom BfS etablierten webbasierten IT-System zu bedeutsamen Vorkommnissen in der Medizin („BeVoMed“). Mithilfe dieses Systems wurden die von den zuständigen Landesbehörden übermittelten Informationen über bedeutsame Vorkommnisse erfasst, verarbeitet und ausgewertet. Das BeVoMed-System wurde seit der Implementierung 2019 bereits mehrfach überarbeitet und bietet aktuell verbesserte Auswertemöglichkeiten nicht nur für das BfS, sondern auch für die jeweils zuständigen Landesbehörden. Für die vorliegende Arbeit wurden alle gemeldeten Vorkommnisse zu Röntgenanwendungen noch einmal im Zusammenhang systematisch im Detail betrachtet. Dies führte insbesondere aufgrund der seit 2019 gesammelten Erfahrung in wenigen Fällen zu einer fachlichen Neubewertung, wodurch die im Folgenden präsentierten Zahlenangaben geringfügig von den in den Jahresberichten 2019–2022 präsentierten Angaben abweichen können.

Ausgewertet wurden alle Meldungen zu diagnostischen und interventionellen Röntgenanwendungen, welche zwischen 01/2019 und 10/2023 beim BfS eingegangen sind und zu denen zum Stichtag, dem 31.10.2023, eine Abschlussmeldung mit Detailinformationen vorlag. Zunächst erfolgte eine Auswertung unter statistischen Gesichtspunkten. Hierfür wurden alle eingegangenen Meldungen hinsichtlich

- ihrer Einordnung als Vorkommnis an sich (§ 1 Abs. 22 StrlSchV),
- ihrer Bedeutsamkeit (§ 108 bzw. Anlage 14 StrlSchV),
- den jeweiligen Meldekriterien der Anlage 14 StrlSchV ( Tab. 1 ) oder als bedeutsam außerhalb von Anlage 14 und
- ihrer Zuordnung zu den jeweiligen Versorgungsformen im deutschen Gesundheitssystem (Praxis, Gemeinschaftspraxis, Krankenhaus der Grundversorgung, Krankenhaus der Regelversorgung, Krankenhaus der Maximalversorgung, medizinisches Versorgungszentrum, andere)

**Table:** **Tab. 1** Für Röntgenanwendungen relevante Kriterien für die Bedeutsamkeit von Vorkommnissen nach Anlage 14 StrlSchV.

**Kategorie I**	**Untersuchungen mit ionisierender Strahlung – ohne Interventionen**	
**Nummer 1**	**Bezogen auf eine Gruppe von Personen**	
**Nummer 2**	**Bezogen auf eine einzelne Person**	
	**a**	CTDI _vol_ einer Computertomografie am Gehirn >120 mGy oder am Körper >80 mGy, DFP einer Röntgendurchleuchtung >20000 cGy cm ^2^
	**b**	Wiederholung einer Anwendung mit Dosisüberschreitung
	**c**	Personenverwechslung mit Dosisüberschreitung
	**d**	Unerwartete deterministische Wirkung
**Kategorie II**	**Interventionen**	
**Nummer 1**	**Bezogen auf eine Gruppe von Personen**	
**Nummer 2**	**Bezogen auf eine einzelne Person, wenn die Intervention zum Zweck der Untersuchung der Person erfolgt**	
	**a**	DFP >20000 cGy cm ^2^
	**b**	Wiederholung einer Anwendung mit Dosisüberschreitung
	**c**	Personenverwechslung
	**d**	Unerwartete deterministische Wirkung
**Nummer 3**	**Bezogen auf eine einzelne Person, wenn die Intervention zum Zweck der Behandlung der Person erfolgt**	
	**a**	Deterministische Hautschäden bei DFP >50000 cGy cm ^2^
	**b**	Personen- oder Körperverwechslung
	**c**	Unerwartete deterministische Wirkung
**Kategorie V**	**Betreuungs- und Begleitpersonen nach § 2 Absatz 8 Nummer 3 StrlSchG**	
**Kategorie VI**	**Anwendung ionisierender Strahlung oder radioaktiver Stoffe am Menschen zum Zweck der medizinischen Forschung**	
**Kategorie VII**	**Ereignisse mit beinahe erfolgter Exposition**	

klassifiziert. Für die als bedeutsam eingestuften Vorkommnisse wurden die genannten Aspekte quartalsweise über den gesamten Auswertezeitraum statistisch analysiert, um die zeitliche Entwicklung seit Einführung der Meldepflicht zu bewerten.

In der anschließenden systematischen Auswertung der Meldungsinhalte wurden gehäuft auftretende Probleme und die zugrundeliegenden Ursachen im Detail identifiziert.

## Ergebnisse

## Statistische Auswertung

Zum Stichtag (31.10.2023) lagen dem BfS insgesamt 383 Erstmeldungen zu diagnostischen und interventionellen Röntgenanwendungen vor, davon waren 355 Meldungen – 278 aus der Röntgendiagnostik und 77 aus der Intervention – abgeschlossen. Bei 82 Meldungen konnte das BfS nicht nachvollziehen, dass es sich überhaupt um ein Vorkommnis handelte; bei 21 Meldungen, dass die Bedeutsamkeit gegeben war. Insgesamt gingen daher 252 bedeutsame Vorkommnisse – 228 zu diagnostischen und 24 zu interventionellen Röntgenanwendungen – in die weitere Auswertung ein ( Tab. 2 ).

**Table:** **Tab. 2** Verteilung aller abgeschlossenen Meldungen (Stand: 31.10.2023) zu diagnostischen und interventionellen Röntgenanwendungen stratifiziert nach den Kriterien der Anlage 14 StrlSchV sowie ihrer Einstufung als Vorkommnis (§1 Abs 22 StrlSchV) und Bedeutsamkeit (§ 108 Abs 1 StrSchV).

Kategorie, Kriterium		Gesamt	Bedeutsam	Nicht bedeutsam	Kein VK
**I. Diagnostik**		**267**	**223**	**15**	**29**
Nr. 1		17	17	–	–
Nr. 2	a)	219	190	–	29
	b)	16	15	1	–
	c)	15	1	14	–
	d)	**–**	–	–	–
Außerhalb Anlage 14		**10**	**5**	**5**	–
**II. Interventionen**		**65**	**18**	**–**	**47**
Nr. 1		1	1	–	–
Nr. 2	a)	55	10	–	45
	b)	–	–	–	–
	c)	–	–	–	–
	d)	–	–	–	–
Nr. 3	a)	7	5	–	2
	b)	–	–	–	–
	c)	2	2	–	–
Außerhalb Anlage 14	CT-Interventionen	11	5	–	6
	Andere	**1**	**1**	–	**–**
**VI. Medizinische Forschung**		**1**	**–**	**1**	**–**
**Gesamt**		**355**	**252**	**21**	**82**
VK: Vorkommnis					

Bezüglich der Röntgendiagnostik zeigte sich bis zum Jahr 2022 ein deutlicher Anstieg der Anzahl an gemeldeten bedeutsamen Vorkommnissen, der sich für das Jahr 2023 fortsetzt, wobei zu beachten ist, dass für 2023 belastbare Zahlen nur bis einschließlich erstes Quartal vorliegen, was sich durch den Meldeverzug aufgrund der Fristensetzung nach § 108 StrlSchV ergibt ( Tab. 3 ). Eine Korrelationsanalyse auf Basis der Quartalsdaten ergab einen schwach positiven aber signifikanten Anstieg. Eine unterjährige Tendenz über die Quartale ist nicht zu erkennen ( Abb. 1 ).

**Table:** **Tab. 3** Verteilung der bedeutsamen Vorkommnisse im Zusammenhang mit diagnostischen und interventionellen Röntgenanwendungen stratifiziert nach den Kriterien der Anlage 14 StrlSchV nach Jahren (Stand: 31.10.2023).

Kategorie, Kriterium		2019	2020	2021	2022	2023*
**I. Diagnostik**		**8**	**35**	**45**	**78**	**57**
Nr. 1		3	7	1	4	2
Nr. 2	a)	5	24	38	69	54
	b)	–	3	6	5	1
	c)	–	1	–	–	–
	d)	–	–	–	–	–
Außerhalb Anlage 14		**–**	**1**	**2**	**1**	**1**
**II. Interventionen**		**1**	**6**	**4**	**6**	**1**
Nr. 1		–	1	–	–	–
Nr. 2	a)	1	2	4	2	1
	b)	–	–	–	–	–
	c)	–	–	–	–	–
	d)	–	–	–	–	–
Nr. 3	a)	–	1	–	4	–
	b)	–	–	–	–	–
	c)	–	2	–	–	–
Außerhalb Anlage 14	CT-Interventionen	**–**	**–**	**1**	**3**	**1**
	Andere	**–**	**–**	**–**	**1**	**–**
**Gesamt**		**9**	**42**	**52**	**89**	**60**
*Bis zum 31.10.2023.						

**Abb. 1:** Quartalsweise Häufigkeit bedeutsamer Vorkommnisse im Zusammenhang mit diagnostischen und interventionellen Röntgenanwendungen entsprechend der Meldekategorien nach Anlage 14 StrlSchV.

Die meisten Meldungen betrafen Überschreitungen der Meldeschwelle bei Einzelpersonen in der CT-Diagnostik (Kategorie I, Kriterium 2a), gefolgt von Überschreitungen bei Gruppen von Patienten durch gleichartige CT-Untersuchungen (Kategorie I, Kriterium 1) sowie Wiederholungen einer Anwendung (Kategorie I, Kriterium 2b). Zu Personenverwechslungen mit einer daraus resultierenden Dosisüberschreitung (Kategorie I, Kriterium 2c) wurde nur eine Meldung abgegeben. Eine deterministische Wirkung, die für die festgelegte Untersuchung nicht zu erwarten war, (Kategorie I, Kriterium 2d) wurde nicht berichtet. Zu interventionellen Röntgenanwendungen gingen insgesamt nur wenige Meldungen ein ( Tab. 3 und Abb. 1 ). Die meisten Vorkommnisse hierbei betrafen Überschreitungen der Meldeschwellen bei Einzelpersonen im Rahmen diagnostischer Interventionen (Kategorie II, Kriterium 2a) oder therapeutischer Interventionen (Kategorie II, Kriterium 3a). Vereinzelte Meldungen betrafen Vorkommnisse, die als bedeutsam außerhalb der Kriterien von Anlage 14 einzuordnen waren ( Tab. 2 und Abb. 1 ). Dies betraf insbesondere Meldungen zu CT-Interventionen.

Bezogen auf die einzelnen Versorgungsformen zeigt sich eine große Diskrepanz hinsichtlich der Meldehäufigkeiten. Zur Röntgendiagnostik gingen die häufigsten Meldungen aus Krankenhäusern der Maximalversorgung ein, gefolgt von Krankenhäusern der Regelversorgung und der Grundversorgung. Diese Versorgungsformen waren auch die einzigen, aus denen Meldungen zu Röntgenintervention erfolgten, wobei auch hier die Häuser der Maximalversorgung dominierten. Aus dem niedergelassenen Bereich gingen die wenigsten Meldungen ein ( Abb. 2 ).

**Abb. 2:** Anzahl an Meldungen zu bedeutsamen Vorkommnissen getrennt nach Versorgungformen im deutschen Gesundheitswesen und Arten von Röntgenanwendungen.

## Systematische Auswertung

Die eingegangenen Meldungen, welche seitens des BfS als kein Vorkommnis eingestuft wurden, betrafen in der Röntgendiagnostik ausschließlich diagnostische CT-Anwendungen an einzelnen Personen, u.a. acht Meldungen zu Perfusions-CT-Untersuchungen des Kopfes in der Schlaganfall-Bildgebung. Als nicht bedeutsam hingegen wurden in der Röntgendiagnostik mit 14 Fällen vor allem Personenverwechslungen ohne entsprechende Überschreitung der Meldeschwelle gewertet (Kategorie I, Kriterium 2c). Bei interventionellen Röntgenanwendungen handelte es sich überwiegend um diagnostische Angiografien an Einzelpersonen, bei welchen trotz Grenzwertüberschreitung keine unbeabsichtigte Exposition vorlag.

Hinsichtlich der bedeutsamen Vorkommnisse ergab sich als ein Problemfeld die fehlerhafte Parameter- oder Protokollwahl, wodurch es zu Überschreitungen der Meldeschwellen kam. Dabei wurden überwiegend patientenspezifische Anpassungen der Standardeinstellungen vorgenommen, welche nicht gerechtfertigt waren. In wenigen Fällen führte auch die automatische Anpassung von Röhrenstrom und -spannung zu einer meldepflichtigen Überexposition. So wurde die systeminhärente Modulation der beiden Parameter beispielsweise durch einen zur Lagerung des Patienten eingesetzten Sandsack oder eine Änderung der Armlagerung nach der Übersichtsaufnahme gestört.

Eine weitere Häufung von bedeutsamen Vorkommnissen betraf Probleme oder Fehler bei der Kontrastmittelgabe. Insbesondere bei CT-Angiografien unter Verwendung eines Bolus-Trackings erfolgte u.a. keine oder eine fehlerhafte Zufuhr des Kontrastmittels, z.B. aufgrund einer defekten Kontrastmittelpumpe, eines unvollständig konnektierten Schlauchs, eines verschlossenen Dreiwegehahns oder Paravasaten. Gemeinsam mit einer nicht optimierten Parameterwahl beim Bolus-Tracking führte die verzögerte oder fehlende Kontrastmittelanflutung und die konsekutiv verlängerte Tracking-Zeit zu einer Überschreitung der Meldeschwelle. Teilweise musste die Untersuchung ein oder sogar mehrfach wiederholt werden, wodurch in der Summe ebenfalls die Meldeschwelle überschritten wurde.

Für (CT-)Intervention ließen sich angesichts der insgesamt noch sehr wenigen Meldungen (außerhalb der Anlage 14 StrlSchV) keine Problemfelder erkennen.

## Diskussion

Seit der Implementierung des Meldesystems für bedeutsame Vorkommnisse ist bezüglich diagnostischer Röntgenanwendungen jährlich ein Zuwachs an Meldungen zu verzeichnen, zu interventionellen Röntgenanwendungen gingen und gehen hingegen nur sporadisch und mit vergleichsweise niedriger Häufigkeit Meldungen ein. Bezogen auf ca. 12 Millionen durchgeführte CT-Untersuchungen in Deutschland pro Jahr 1 wären insgesamt weitaus mehr bedeutsame und damit meldepflichtige Vorkommnisse zu erwarten. Ein vom BfS konzipiertes und betreutes Forschungsvorhaben aus dem Jahr 2018, welches 2020 beendet wurde, ergab, dass allein aus dem Krankenhaussektor geschätzt etwa 1000 bedeutsame Vorkommnisse zu beiden Anwendungsarten pro Jahr zu erwarten wären 5 .

Andererseits gab es eine Vielzahl gemeldeter Ereignisse, die aus Sicht des BfS entweder als kein Vorkommnis (§ 1 Abs. 22 StrlSchV) oder als nicht bedeutsam (§ 108, Anlage 14 StrlSchV) einzuordnen sind. Beispielsweise wurden bei CT-Untersuchungen adipöser Personen geplant erhöhte Dosen appliziert, um die notwendige diagnostische Bildqualität zu erreichen, oder es wurden bei komplexen angiografischen Interventionen zum Erreichen des diagnostischen oder therapeutischen Ziels eine Überschreitung der Meldeschwelle bewusst akzeptiert. Daraus lässt sich schließen, dass es seitens der SSV noch viele Unklarheiten sowohl hinsichtlich der Definition eines Vorkommnisses als auch dessen Bedeutsamkeit gibt. Um als Vorkommnis gewertet zu werden, muss ein Ereignis mit einer unbeabsichtigten Exposition einhergehen, d.h. mit einer Exposition, die nicht im Rahmen der individuellen rechtfertigenden Indikation prospektiv geplant war. Somit liegt bei einer *prospektiv* vorgesehenen Anwendung mit entsprechender rechtfertigender Indikation (§ 83 Abs. 3 StrlSchG) und regelgerechtem Vorgehen während der Durchführung kein Vorkommnis vor, wenn die jeweilige Meldeschwelle, z.B. aufgrund der Patientenstatur, überschritten wurde. Dies betrifft insbesondere Meldungen zu diagnostischen Interventionen, bei denen zwar eine erhöhte, aber als beabsichtigt anzusehende Strahlenexposition beschrieben wurde.

Auch hinsichtlich der Bedeutsamkeit von Vorkommnissen scheinen weiterhin Missverständnisse und Unklarheiten zu bestehen. Da § 108 StrlSchV hierzu „insbesondere“ auf die Kriterien der Anlage 14 StrlSchV verweist, sind diese als nicht abschließende Auflistung zu betrachten. Daraus ergibt sich ein gewisser Spielraum für die Bewertung eines Vorkommnisses, für welches kein explizites Meldekriterium in Anlage 14 genannt ist. So sollten beispielsweise auch Vorkommnisse als bedeutsam gemeldet werden, deren Wiederholung durch andere Anwendende oder bei ähnlichen Anwendungen durch entsprechende Hinweise vermieden werden könnten. Das BfS hat zur Klärung der häufigsten Fragen (FAQ) bezüglich der Interpretation von Anlage 14 StrlSchV eine entsprechende Webseite eingerichtet 6 .

Bedeutsame Vorkommnisse bei Röntgenuntersuchungen betrafen hauptsächlich die CT-Diagnostik bei Einzelpersonen (Kategorie I, Kriterium 2a), gefolgt von CT-Untersuchungen bezogen auf eine Gruppe von Personen (Kategorie I, Kriterium 1). So wurden beispielsweise bei 25 Personen CT-Untersuchungen der knöchernen Lendenwirbelsäule mit einem Protokoll durchgeführt, welches für die Untersuchung der Bandscheiben vorgesehen war 7 . CT-Untersuchungen erfolgen einerseits sehr häufig und werden andererseits aufgrund ihrer relativ hohen Expositionen explizit in der Anlage 14 StrlSchV berücksichtigt, wohingegen die ebenfalls sehr häufigen Anwendungen der Projektionsradiografie und der digitalen Volumentomografie der Zähne und des Kiefers aufgrund der mit ihnen verbundenen niedrigen Expositionen ausgenommen sind (siehe Begründung der StrlSchV 8 sowie für weiterführende Erläuterungen Brix et al. 2 ).

Aus dem interventionellen Bereich gingen die meisten Meldungen zu diagnostischen Angiografien gefolgt von vaskulären Interventionen ein. Da für die Bedeutsamkeit von Vorkommnissen bei CT-Interventionen bislang keine Kriterien in Anlage 14 festgelegt sind, liegt die Entscheidung, ob ein solches Vorkommnis als bedeutsam einzustufen ist, im jeweiligen Einzelfall im Ermessen des/der SSV – idealerweise nach Rücksprache mit der zuständigen Landesbehörde.

Die meisten Meldungen aus der Röntgendiagnostik gingen aus Krankenhäusern der Maximalversorgung ein. Dies ist dadurch zu erklären, dass in diesen Häusern einerseits häufig komplexe Strahlenanwendungen in Diagnostik und Therapie durchgeführt werden und andererseits zumeist umfassende Qualitätssicherungssysteme etabliert sind. Aus dem niedergelassenen Bereich hingegen wurde, sowohl für diagnostische als auch für interventionelle Röntgenanwendungen, nur sehr spärlich gemeldet. Für Interventionen ist dies darauf zurückzuführen, dass diese insgesamt seltener ambulant durchgeführt werden. Dennoch wären auch aus diesem Bereich deutlich mehr Meldungen zu erwarten. Darüber hinaus könnte dies auf eine bisher noch unzureichende Rezeption des neuen Strahlenschutzrechts und ein konsekutiv fehlendes Bewusstsein für die Meldepflicht hindeuten.

Die Analyse der Meldeinhalte ergab ein Problemfeld bei der Anwahl geeigneter Protokolle und der Einstellung optimaler Protokollparameter für die jeweilige Untersuchung. Unbedarfte Anpassungen von Röhrenstrom und -spannung können zu erhöhten Dosisexpositionen mit Überschreitung von Meldeschwellen führen. Bei einer insuffizienten Voreinstellung, insbesondere bei inadäquat hinterlegten Protokollen, können auch mehrere Personen nacheinander von erhöhten Expositionen betroffen sein. Um dies zu vermeiden, besteht die Notwendigkeit, hinterlegte Protokolle regelmäßig zusammen mit einem/einer Medizinphysikexpert*in (vgl. § 131 StrlSchV) zu überprüfen. Grundsätzlich sind Dosismanagementsysteme ein geeignetes Instrument, um nicht gerechtfertigte Expositionen zu erkennen.

Die beobachtete Häufung von Vorkommnissen im Zusammenhang mit der Kontrastmittelgabe bei CT-Untersuchungen resultiert größtenteils aus der Verwendung von Bolus-Tracking-Verfahren. So können bei verzögerter oder fehlender KM-Anflutung und dadurch verlängerter Tracking-Zeit unbeabsichtigt CTDI _vol_ -Werte von mehr als 80 mGy erreicht werden, welche meldepflichtig sind. Zwar ist die strahlenhygienische Relevanz einer derartigen Exposition in einer einzelnen oder in wenigen CT-Schichten nicht vergleichbar mit einer Überschreitung des CTDI _vol_ -Werts über einen größeren Körperbereich, wie beispielsweise dem Abdomen. Dennoch gilt auch in den genannten Fällen das Optimierungsgebot sowohl hinsichtlich des eingesetzten Röhrenstroms als auch insbesondere der Bildwiederholrate und der Trackingdauer. Auch ist im Sinne des Strahlenschutzes kritisch zu hinterfragen, ob bei jeder Untersuchung die Anwendung des Bolus-Trackings notwendig ist. Dies sollte im Rahmen der zu stellenden rechtfertigenden Indikation berücksichtigt werden.

Neben der Etablierung einer positiv verstandenen und schamfreien Fehlerkultur in klinischen Einrichtungen ist aus Sicht des BfS ein funktionierendes lokales Qualitätsmanagement, das die Anforderungen von § 105 StrlSchV erfüllt, die Grundvoraussetzung für die Optimierung von Abläufen. Dabei sind Maßnahmen wie die Implementierung von Dosismanagementsystemen oder die Erstellung von Arbeitsanweisungen hilfreich. Ebenso ist, wie bereits erwähnt, eine regelmäßige Protokolloptimierung an den eingesetzten Geräten anzuraten. Erfordert die jeweilige klinische Situation eine – im Einzelfall gegebenenfalls durchaus gerechtfertigte – Abweichung von festgelegten Arbeitsabläufen und Protokollen, so sollte das daraus resultierende erhöhte Risikopotenzial berücksichtigt werden. Im Sinne der Patient*innensicherheit ist daher sowohl eine ausreichende Personalausstattung als auch die Funktionsfähigkeit adäquater Sicherheitsvorkehrungen zu gewährleisten 9 . Die Entwicklung der Meldezahlen seit Einführung einer Meldepflicht für bedeutsame Vorkommnisse lässt auf die zunehmende Etablierung von Qualitätssicherungssystemen zur Erkennung und Vermeidung von Vorkommnissen auf lokaler Ebene als auch ein zunehmendes Problembewusstsein sowie der Akzeptanz der Meldepflicht schließen. Hierbei spielt sicherlich auch die nun verpflichtende Mitarbeit von Medizinphysikexpert*innen (§ 131 Abs. 2 Nr. 3 und 4 StrlSchV) eine Rolle, welche inzwischen nach Ablauf der Übergangsfrist (§§ 198 und 200 StrlSchG) auch für Bestandsgeräte gilt.

Im internationalen Vergleich liegt die Meldefrequenz bedeutsamer Vorkommnisse in Deutschland, bezogen auf die Einwohnerzahl, hinter derjenigen in Irland 10 , Australien 11 oder der Schweiz 12 . Da sich in diesen Ländern jedoch sowohl die Kriterien für die Bedeutsamkeit von Vorkommnissen als auch die Organisation der Gesundheitssysteme von den Gegebenheiten in Deutschland unterscheiden, ist ein direkter Vergleich nur eingeschränkt möglich. Auch in genannten Ländern wurden jedoch am häufigsten Vorkommnisse im Zusammenhang mit der CT-Diagnostik gemeldet. Zu erhöhten Expositionen kam es in Irland überwiegend durch menschliches Fehlverhalten, in Australien durch unnötige oder unbeabsichtigte Untersuchungen und durch technische Gerätefehler sowie in der Schweiz durch Personenverwechslungen, falsche Untersuchungsprotokolle oder ungewollte Wiederholungen der Untersuchung. Aus dem interventionellen Bereich gingen, ähnlich wie in Deutschland, jeweils wenige Meldungen ein. Die australische Strahlenschutzbehörde beschreibt u.a. gemeldete Angiografien mit erhöhter Dosisexposition aufgrund nicht beeinflussbarer Gegebenheiten, z.B. Komplexität oder Adipositas.

Nach wie vor gibt es sowohl seitens der SSV als auch der zuständigen Länderbehörden Unklarheiten bezüglich der Definition von Vorkommnissen und deren Bedeutsamkeit. Der vorgegebene Meldeweg in Deutschland sieht vor, dass der oder die jeweilige SSV ein bedeutsames Vorkommnis an die zuständige Behörde meldet, welche die Meldungen bewertet und im Falle eines bedeutsamen Vorkommnisses fachlich relevante Informationen an das BfS übermittelt. Da diese Behörden jeweils einen gewissen Ermessensspielraum haben, und es sich nicht bei jedem gemeldeten Ereignis wirklich um ein Vorkommnis handelt bzw. nicht jedes gemeldete Vorkommnis bedeutsam ist, kann es zu Unterschieden zwischen der Anzahl der von den SSV abgegebenen und der bei dem BfS eingegangenen Anzahl an Meldungen kommen. Da darüber hinaus das Meldesystem im Rahmen der strahlenschutzrechtlichen Aufsicht implementiert wurde, kann nicht ausgeschlossen werden, dass Meldungen aus Angst vor negativen Konsequenzen unterbleiben. Zu beiden Sachverhalten liegen dem BfS systemimmanent leider keine konkreten Zahlen vor.

Zusammenfassend zeigt das Meldesystem zu bedeutsamen Vorkommnissen bei Strahlenanwendungen am Menschen bei Röntgenanwendungen eine positive Entwicklung über die letzten Jahre, was von einer zunehmenden Fehlerkultur zeugt. Es scheinen jedoch noch Unklarheiten in Bezug auf die Einordnung eines Ereignisses als Vorkommnis und dessen Bedeutsamkeit vorzuliegen. Es sollte das gemeinsame Ziel aller Beteiligten sein, die Sicherheit von Strahlenanwendungen in Deutschland kontinuierlich weiter zu verbessern. Hierzu ist die kontinuierliche Verbesserung und Stärkung eines adäquaten Problembewusstseins und einer entsprechenden Fehlerkultur über alle Berufsgruppen hinweg von zentraler Bedeutung.
